# 3D Graphene Scaffolds for Skeletal Muscle Regeneration: Future Perspectives

**DOI:** 10.3389/fbioe.2020.00383

**Published:** 2020-05-05

**Authors:** Valentina Palmieri, Francesca Sciandra, Manuela Bozzi, Marco De Spirito, Massimiliano Papi

**Affiliations:** ^1^Dipartimento di Neuroscienze, Università Cattolica del Sacro Cuore, Rome, Italy; ^2^Fondazione Policlinico Universitario Agostino Gemelli IRCCS, Rome, Italy; ^3^Istituto di Scienze e Tecnologie Chimiche “Giulio Natta”, (SCITEC)-CNR, SS Roma, Italy; ^4^Dipartimento di Scienze Biotecnologiche di Base, Cliniche Intensivologiche e Perioperatorie, Sezione di Biochimica e Biochimica Clinica, Università Cattolica del Sacro Cuore, Rome, Italy

**Keywords:** graphene, scaffold, skeletal muscle, tissue engineering, implants

## Abstract

Although skeletal muscle can regenerate after injury, in chronic damages or in traumatic injuries its endogenous self-regeneration is impaired. Consequently, tissue engineering approaches are promising tools for improving skeletal muscle cells proliferation and engraftment. In the last decade, graphene and its derivates are being explored as novel biomaterials for scaffolds production for skeletal muscle repair. This review describes 3D graphene-based materials that are currently used to generate complex structures able not only to guide cell alignment and fusion but also to stimulate muscle contraction thanks to their electrical conductivity. Graphene is an allotrope of carbon that has indeed unique mechanical, electrical and surface properties and has been functionalized to interact with a wide range of synthetic and natural polymers resembling native musculoskeletal tissue. More importantly, graphene can stimulate stem cell differentiation and has been studied for cardiac, neuronal, bone, skin, adipose, and cartilage tissue regeneration. Here we recapitulate recent findings on 3D scaffolds for skeletal muscle repairing and give some hints for future research in multifunctional graphene implants.

## Introduction

The discovery of methods to generate and genetically manipulate stem cells, the advances in bio-fabrication technologies including 3D bioprinting and the innovations biomimetic biomaterials are the three pillars of modern tissue engineering (Khademhosseini and Langer, 2016). The bidimensional flake of carbon, graphene, represents undoubtedly the revolutionary material of the last decade. Graphene has a hexagonal lattice structure of sp2 hybridized carbon atoms and is extremely thin (<0.5 nm), electrically and thermally conductive, mechanically resistant and light absorptive (Dreyer et al., 2014; Trusovas et al., 2016). Its derivative, the graphene oxide (GO) has oxygen functional groups decorating carbon plane and has largely been exploited in medicine due to its water dispersibility. Indeed pristine graphene is highly hydrophobic and tends to precipitate in biological media (Huang et al., 2020). GO has been functionalized with polymers and biomolecules and is also the precursor of rGO, the reduced form obtained by different chemical, hydrothermal, electrochemical procedures and similar to pristine graphene, but with defects and holes on the carbon skeleton (Dreyer et al., 2014; Palmieri et al., 2019a, b). Graphene-based materials (GBM) have been studied for several applications in biomedicine due to their unique interactions with proteins and molecules: from the first studies, it was evident that the surface area and chemistry of GBM allowed high adsorption of proteins that can mediate interactions with cells, bacteria and therapeutic compounds that can be delivered by graphene nanoflakes (Palmieri et al., 2018; Di Santo et al., 2019; Papi et al., 2019). Several methods to produce 3D graphene scaffolds have been designed, from hydrogels to electrospun graphene fibers and 3D printed GBM scaffolds (Zhang et al., 2018; Choe et al., 2019a; Li et al., 2019; Palmieri et al., 2020).

Graphene-based materials have been largely 3D printed or bioprinted (Palmieri et al., 2020). Though studies on 3D-printed GBM for cardiac muscles have been conducted (Choe et al., 2019a; Li et al., 2019), there is a lack of production of 3D printed GBM scaffolds for skeletal muscle regeneration. In this mini-review, we want to define a primer to boost research on 3D printed implants for skeletal muscles based on GBM (Zhang et al., 2018). We foresee that graphene multi-functional scaffolds will represent the future of myo-regeneration based on 3D scaffolds.

## Repairing Skeletal Muscle

**FIGURE 1:**
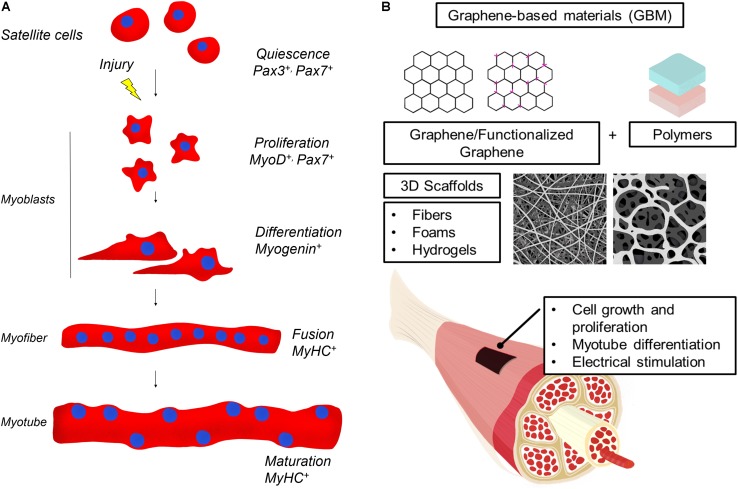
**(A)** Skeletal muscle regeneration. Upon injury, quiescent muscle stem cells (satellite cells) undergo rapid proliferation, followed by differentiation into myoblasts, which fuse and mature to generate new muscle fibers. Muscle regeneration is characterized by the activation of transcription factors that are specific for each stage of the myofiber maturation process. In the figure, the most relevant modulators of myogenic lineage progression are indicated. **(B)** Illustration of GBM materials usage in skeletal muscle regeneration. GBM are obtained by combining graphene or graphene derivatives with natural or artificial polymers and a 3D scaffold is produced. GBM effects on muscle cells include induction of cell proliferation and differentiation. GBM are also conductive and can be electrically stimulated to favor muscle regeneration.

Skeletal muscle tissue is composed of multinucleated contractile muscle cells, the myofibers (Figure 1A). Parallel-aligned myofibers are bundled together to form fascicles and multiple fascicles are held by connective tissue to form mature muscle. Skeletal muscle can efficiently repair itself in response to injury (Tedesco et al., 2010). Muscle regeneration is a highly coordinated process that requires the recruitment of a pool of undifferentiated cells, called satellite cells. Satellite cells normally lie in a quiescence state beneath the basal lamina of muscle fibers but upon muscle injury are induced to proliferate, fuse and differentiate into multinucleated fibers leading to the complete regeneration of the injured muscle (Schultz and McCormick, 1994). Immediately after a muscle injury, proteolytic enzymes, cytokines and growth factors are released creating a local microenvironment that stimulates the migration of inflammatory cells and fibroblasts at the site of injury. A new temporary extracellular matrix (ECM) is produced stabilizing the tissue and acting as a scaffold to direct the migration of the satellite cells in the injured site (Charge and Rudnicki, 2004). Satellite cells are induced to differentiate in myoblasts that then fuse in mature myotubes. It is through this combination of ECM with muscle fibers that the injured muscle is repaired. This regeneration phase peaks at 2 weeks after injury and it also involves the generation of new blood vessels and nerves.

However, in chronic tissue damage, such as muscular dystrophies, or in acute severe muscle tissue loss due to traumatic injuries, skeletal muscle is unable to fully regenerate its structure leading to functional impairment and severe disability (Liu et al., 2018). Current cell therapies characterized by locally or systematically injection of stem cells or other myogenic/non-myogenic cells are promising (Tedesco and Cossu, 2012) however, poor cell survival remains a challenge. GBM-scaffolds provide a structural synthetic framework that recreates the tridimensional microenviroment favorable for cell adhesion and proliferation (Jenkins and Little, 2019). *In vitro* analysis of these engineered muscles envisages morphological and functional evaluation of muscle maturation (Juhas et al., 2016), but the large-scale production of scaffolds induced the development of computational modeling systems for high-throughput testing (Torii et al., 2018).

## 3D Graphene Scaffolds and Myogenesis

For skeletal muscle regeneration, scaffolds need particular features to direct fusion of myocytes in multinucleated myotubes, stimulate vascularization and innervations. Moreover, materials have to degrade in a biocompatible way when the regenerated tissue is stable (Grasman et al., 2015; Torii et al., 2018). In Table 1, we summarize GBM 3D scaffolds produced and, in the following paragraphs, we analyze the advantages of using GBM scaffolds (Figure 1B).

**TABLE 1:** 3D graphene scaffolds for myoblasts growth and differentiation.

	GBM composite	Cells	Effects	References
Self-assembling composites	GO-Gelatin	C2C12	Good cell growth and proliferation within the hydrogel and spontaneous myogenic differentiation	Lee et al., 2016
	Graphene-CS-GG	C2C12	Efficient cell spreading, proliferation and differentiation	Patel et al., 2018
	GO-Alginate	C2C12	Increased viability of cells, for *in vivo* cell delivery	Ciriza et al., 2015
	GO-(PAAm); rGO-(PAAm)	C2C12	Improved myoblast growth, myogenic differentiation and electrical stimulation on rGO-(PAAm) compared to GO-(PAAm), alignment along micropatterns	Jo et al., 2017; Park et al., 2019
	rGO-PCEG	C2C12 and *in vivo*	Enhanced proliferation, differentiation and formation of muscle fibers and blood vessels *in vivo*	Du et al., 2018
Foams	Graphene-laminin	C2C12	Efficient differentiation into myotubes and contraction upon electrical stimulus	Krueger et al., 2016
	GO-PU	C2C12	Promoted myogenic differentiation	Shin et al., 2018a
Electrospun fibers	GO-RGD-PLGA	C2C12	Enhanced adhesion, proliferation, myoblasts alignment and fusion	Shin et al., 2018b
	GO-PLGA-Collagen	C2C12	Enhanced attachment, proliferation and differentiation	Shin et al., 2015
	GO-PCL	C2C12; CB-hSkMCs	Promoted myogenic differentiation and activation of IGF signaling	Chaudhuri et al., 2014, 2016
	Graphene-PCL	C2C12	Promoted adhesion proliferation and differentiation in growth media	Patel et al., 2016
	GO-PU	C2C12	Enhanced initial adhesion and spreading, up-regulated the myogenic mRNA levels and myosin heavy chain expression. Mechanically stretchable fibers	Bin Jo et al., 2020

*CB-hSkMCs, human cord blood mesenchymal stem cells derived skeletal myoblast; CS, chitosan; GG, gellan gum; GO, graphene oxide; rGO, reduced graphene; PAAm, polyacrylamide; PCEG, Poly (citric acid-octanediol-polyethylene glycol); PCL, poly(ε-caprolactone); PLGA, poly(lactic-co-glycolic acid); PU, polyurethane; RGD, Arg-Gly-Asp peptide.*

### Graphene Composites

Graphene composites for 3D scaffolds production are usually synthesized by combining a GBM and a synthetic or natural polymer. Natural biomaterials are usually biocompatible, easy to functionalize and primed for enzymatic degradation (Del Bakhshayesh et al., 2019). However, they are limited by batch-to-batch variability in chemical composition, and some components may be immunogenic. Synthetic polymers, on the other hand, are precisely mechanically and chemically controlled but degradation into byproducts and inflammatory responses might occur in response to this kind of material (Nakayama et al., 2019).

Graphene-based materials bring several advantages in skeletal muscle engineering. First, graphene derivatives can reinforce bare material mechanical properties and simulate muscle response to tension (Rogers and Liu, 2013; Hwang et al., 2019). GO films can mimic the adaptive behaviors of natural muscles including strengthening in response to strain (Dai et al., 2016) much more than other carbon nanomaterials. Natural polymers, like gelatin, collagen and chitosan, combined to GO can induce spontaneous differentiation of C2C12 murine myoblasts cells (Shin et al., 2015; Lee et al., 2016; Patel et al., 2018). Graphene can also enhance materials biocompatibility. GO improves biocompatibility of microbeads of alginate for delivery of encapsulated C2C12 myoblasts (Ciriza et al., 2015) especially if embedded with a protective protein corona that mitigates *in vivo* foreign response (Ciriza et al., 2018). rGO-alginate hydrogels protect mesenchymal stem cells thanks to reactive oxygen species scavenging (Choe et al., 2019a). GO-alginate is bioprintable and bioconductive effects have been observed in many tissue engineering applications, paving the way for skeletal muscle reconstruction studies (Choe et al., 2019b).

As for neurons, electrical conductivity is essential for muscle cells that have indeed the ability to undergo depolarization and repolarization during cell contraction. The main advantage of electrical (and mechanical) stimulation *in vitro* for skeletal muscle cells is the mimicking of the physical simulation of stretch and electrical coupling in muscle. Ion channels on cell membranes generate an electrical potential within the membrane and application of electrical fields can alter the level of intracellular calcium content and cause signal transduction. This occurs *in vivo* for the regulation of muscular function by motor neurons and influences also cell attachment and proliferation. Electrical stimulation is known to cause myogenic differentiation and increase muscular forces (Nakayama et al., 2019). However, the exact mechanism that relates electrical stimulation and myogenic differentiation is still unknown though researchers hypothesized an involvement of calcineurin pathway mediated by the ion flow through cell membrane (Dong et al., 2019). rGO is known to have better electrical conductivity that can be further improved if other materials of the carbon family are anchored to its surface, like carbon nanotubes (Morimoto et al., 2016; Kim et al., 2017). A direct comparison of effects of rGO (obtained by hydrazine reduction) and GO substrates on C2C12 cells was reported in an early study of Ku and Park (2013) with a better performance of GO in the induction of myogenic protein expression, multinucleate myotube formation, and expression of differentiation-specific genes (MyoD, myogenin, Troponin T, and MHC). Nanotopography-mediated cell adhesion cannot explain differences between GO and rGO, that have similar roughness. However, the pro-differentiation effects of GO could be ascribed to its higher ability to adsorb serum proteins through its functional oxygen groups and ability to retain 3D conformation of proteins especially fibronectin, which is a mediator of muscle cell adhesion and differentiation (Ku and Park, 2013). Oppositely, rGO (obtained by ascorbic acid reduction) and polyacrylamide (PAAm) induce a significantly enhanced proliferation and myogenic differentiation compared with GO/PAAm and are electronically stimulable (Jo et al., 2017). These contrasting results could be explained by the mild reduction method employed by authors based on ascorbic acid, which possibly leaves a percentage of oxygen groups that might facilitate protein adsorption and consequently cell attachment. Alternatively, electrically conductive environments could *per se* facilitate electrical communication among muscle cells and result in the induction of increased myogenic differentiation besides external electrical stimulation. Poly (citric acid-octanediol-polyethylene glycol)-graphene (PCEG) composites have been produced and tested *in vivo* in rats as biodegradable and electrically conductive scaffolds. After subcutaneous implantation in rats, there was a lack of immunoreaction and a good capillary formation in the skeletal muscle lesion (Du et al., 2018).

### Graphene Foams

Graphene foams 3D architectures consist of an interconnected lightweight continuous network of graphene sheets and has been used as an effective reinforcing agent in composites for biomedical and electronics (Idowu et al., 2018). Krueger et al. (2016) demonstrated that nickel/graphene foams can induce myotube formation if C2C12 cells are seeded on it, especially if the foam is coated with laminin. Compared to C2C12 cells cultured on planar graphene, the foams exhibited higher cell and myotube densities and have also been successfully used for electrical stimulation (±10 V, 50 ms duration, 1 Hz) and induction of contraction of myotubes (Krueger et al., 2016).

Foams have been also produced by adding GO to polyurethane (PU) and a spontaneous myogenic differentiation of myoblasts ascribed to the synergistic effects of GO and to the “community effect” was observed. This effect occurs when cells grown in the interconnected GO-PU foam pores, having an average size of 300 μm, have an augmented communication among the neighboring cells through cell-cell and cell-scaffold interactions (Shin et al., 2018a).

### GBM Topographies and Electrospun GBM Fibers

Cell alignment is one of the most critical factors in skeletal muscle regeneration. Alignment can be obtained by both simulation of ECM topography and usage of fibrous elements within scaffolding materials (Grasman et al., 2015). Though the exact mechanism through which cells respond to topography is not well understood, it was shown that a period of 6 μm is optimal for myoblast differentiation of myoblasts (Lam et al., 2006). While micropatterning has proven to be efficient in providing contact guidance to alignment, ECM proteins have nanoscale features that stimulate cytoskeletal-responsive pathways to enhance differentiation. Several bioengineering techniques aim to mimic the microenvironment topography features such as ripple and wrinkles that offer contacts for cell adhesion and can enhance stem cell differentiation (Grasman et al., 2015).

Graphene can modify micro and nano-features of 3D scaffolds. For example, the introduction of GO in hydrogel is used to increase the surface roughness (Zhou et al., 2018). Further, compressive strain-induced deformation of graphene substrates has been employed to form crumpled folds and cause the alignment and elongation of myoblasts (Kim et al., 2019). Nanotopographies can be added to surfaces of GBM-containing scaffolds also through laser printing by exploiting light absorption properties of graphene (Papi et al., 2016; Palmieri et al., 2017). Park et al. (2019) used femtosecond laser ablation on hydrogels of GO and PAAm to evaluate the effects of stripes on myoblasts alignment. Micro-groove patterns (20 μm wide and 10 μm deep) were designed on GO scaffolds at 20, 50 or 80 μm distance and then the scaffold was submerged in ascorbic acid to obtain rGO and enhance electrical properties of the samples (Park et al., 2019). rGO/PAAm with a 50 μm pattern showed the best performance for differentiation and myotube alignment and, after the electrical stimulation of myoblasts, the differentiation was further enhanced. These implants were also biocompatible *in vivo*, i.e., didn’t cause recruitment of inflammation cells (Park et al., 2019).

Electrospinning is a versatile technique to produce polymer nanofibers (diameter from 40 to 2000 nm) forming 3D scaffolds and replicate aligned muscle architecture. Electrospinning is performed when the electric force of the mother liquid surface exceeds the surface tension and initiates an electric spark provoking the solution to be ejected from a syringe, and as jet flows the nanofiber is produced. Graphene gained significant interests for electrospinning researchers, for its high strength, flexibility, optical transparency, and conductivity (Javed et al., 2019; Parlayıcı et al., 2019). Electrospinning fibers have been also used for muscle cells growth in several recent papers. GO-poly(ε-caprolactone (PCL) composites have been produced by Chaudhuri et al. (2016) to create a mesh with ∼85% porosity for C2C12 cells growth. On GO-PCL, the myogenic proteins expression (Desmin and MyoD) and cell signaling were improved with a superior myogenic differentiation on these scaffolds, probably due to enhanced conductivity of the GO containing mesh. The same authors demonstrated that also human skeletal muscle cells derived from umbilical cord blood-derived mesenchymal stem cells, can form myotubes on GO-PCL (Chaudhuri et al., 2014). GO-PCL seems to have an effect on the insulin-like growth factor-1 pathway which is related to the myotube formation and maturation (Chaudhuri et al., 2016). Also pristine graphene has been electrospun with PCL. Graphene-PCL had a decrease in impedance with the increase of graphene concentration (up to 2%) and a good cytocompatibility besides being able to induce myogenic differentiation (Patel et al., 2016). The incorporation of GO in electrospun PU improved hydrophilicity, elasticity, and stress relaxation capacity (Bin Jo et al., 2020). PU/rGO composite nanofibrous electrospun scaffolds have been produced for cardiac tissue engineering. The rGO increased the electrical conductivity, Young’s modulus and ultimate tensile strength and decreased the elongation at break of PU. On these scaffolds, troponin I gene expression was enhanced, especially when fibers were produced in aligned arrangement (Azizi et al., 2019). Electrospun fibers of poly(lactic-co-glycolic acid, PLGA) and collagen with GO (GO-PLGA-Col) significantly improved the attachment and proliferation of the C2C12 and stimulated the myogenic differentiation (Shin et al., 2015). Similarly, scaffolds of GO-PLGA enriched with RGD peptide (Arg-Gly-Asp) prompted myoblasts growth and differentiation (Shin et al., 2018b).

### 3D Printing in Skeletal Muscle Research

Despite 3D printing has been successfully employed for the production of skin, adipose, bone and cardiac muscle (Li et al., 2019), limited research on skeletal muscle has been undertaken. 3D printed disks of polylactic acid are capable of myogenic differentiation induction thanks to cell proximity in printed channels (Rimington et al., 2017). PU bioink with muscle cells can be co-printed with fibroblast-containing PCL to obtain differentiated stiffness and also stimulate tissue development and differentiation (Merceron et al., 2015; Choi et al., 2016). Interestingly both PU and PCL have been successfully combined to graphene derivatives in other studies and future research could be focused on muscle-tendon units improved on GBM scaffolds (Shin et al., 2018a; Palmieri et al., 2020).

**FIGURE 2:**
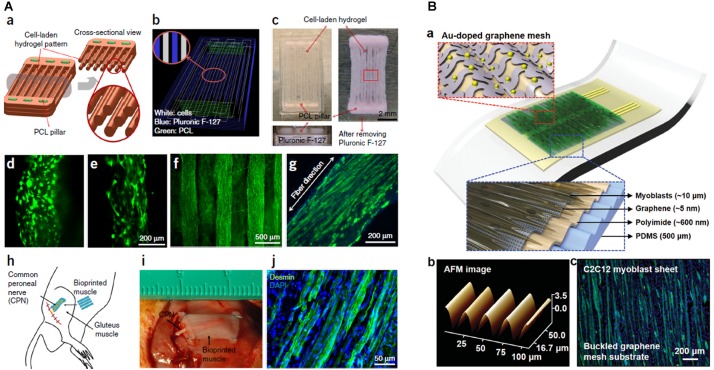
**(A)** Bioprinted muscle. **(a)** Fiber bundle structure for muscle organization with PCL pillars (green) used to maintain the structure and to induce cell alignment. **(b)** Visualized motion program for 3D printing muscle construct. Lines of green, white and blue indicate the dispensing paths of PCL, cell-laden hydrogel and sacrificial material, respectively. **(c)** 3D patterning outcome of designed muscle organization before and after removing the sacrificial material. The PCL pillar structure is essential to stabilize the 3D printed muscle as visible from scaffolds without PCL pillar **(d)** and with PCL pillar **(e)**. The cells with PCL pillar showed unidirectionally organized cellular morphologies **(f)**. The live/dead staining of the encapsulated cells in the fiber structure indicates high cell viability (green: live; red: dead). **(g)** Immunofluorescent staining for myosin heavy chain of the 3D printed muscle organization after 7 days differentiation. **(h)** Schematic diagram of ectopic implantation of bioprinted muscle construct *in vivo*. **(i,j)** The bioprinted muscle construct subcutaneously implanted and the harvested implants after 2 weeks of implantation showed the presence of organized muscle fibers and innervating capability within the implanted construct, as confirmed by desmin muscle marker **(j)**. Reproduced with permission from Kang et al. (2016). Copyright 2016 Nature America. **(B)** The architecture of the stretchable and transparent cell-sheet–graphene hybrid made of layers of C2C12 myoblasts, an ultrathin graphene mesh doped with Au, Au nanomembrane connective electrodes, polyimide and a PDMS substrate **(a)**. Atomic force microscope image of graphene mesh **(b)** and C2C12 myoblasts **(c)** labeled for myosin heavy chain (green) and nuclei (blue). Reproduced with permission from Kim et al. (2016), Copyright Elsevier Ltd., 2019.

Kang et al. (2016) created an integrated tissue-organ, printed and fabricated 3D muscle construct containing mouse myoblasts (Figure 2A). After 2 weeks, the retrieved muscle constructs showed well-organized muscle as well as nerve contacts and vascularization. Similarly, PEG/fibrinogen/alginate constructs generate a fully maturated muscle-like tissue (Costantini et al., 2017).

3D printing has been employed to create artificial muscles that can be moved thanks to the photothermal properties of graphene that can be embedded in prosthesis and allow performing complex motions driven by laser light stimulation (Peele et al., 2015; Han et al., 2019; Helps et al., 2019; Zhou et al., 2019).

## Discussion

The studies reported in this mini-review demonstrated how scaffolds of GBM can induce myogenic differentiation. Additionally, GBM has been also used as a platform for neural cell growth, osteogenic differentiation, and chondrogenic differentiation, the major components of the musculoskeletal system. Besides being electrically activable, GBM have the intrinsic capability of (i) improve the viability of myoblasts (Ciriza et al., 2015) [and more generally of cells attached on surface, thanks to the adsorption of biomolecules and surface roughness (Zhou et al., 2018)] and (ii) induce myogenic differentiation probably facilitating the communication between cells (Jo et al., 2017). GBM have also been used in 2D films for muscle cell growth (Zhang et al., 2018), but we focused on 3D structures since these kinds of architectures recapitulate the native tissue inducing the expression of key myogenic modulators, parallel growth of muscle fibers and a correct organization of cytoskeleton and cell junctions increasing the community effect (Shin et al., 2018a; Naik et al., 2019). Interestingly, 3D printing of GBM has not been exploited for myogenesis, despite graphene inks and filaments are available. GBM can be useful for their electronic properties: GO and rGO have indeed been employed to create multifunctional stretchable and transparent devices implantable *in vivo* for electrostimulation and continuous monitoring of muscles (Figure 2Bb; Kim et al., 2016). Graphene layers have been transferred to 3D printed scaffolds of poly (methyl methacrylate) to improve the conductivity of the polymer (Kim et al., 2020) with possible applicability in muscle regeneration. Besides the stimulation of myoblasts, *in vivo* implantation of graphene-based devices allows also neo-angiogenesis and this property should be exploited in the future also in skeletal muscle research (Kim et al., 2016; Du et al., 2018). GBM are indeed known to be capable of initiation of neurogenesis and neo-vascularization (Li et al., 2019), paramount to prevent atrophy and tissue necrosis (Gilbert-Honick and Grayson, 2020). Traumatic musculoskeletal injuries are accompanied by loss of blood supply and denervation and GBM might fulfill multiple functions as long as the scaffold is engineered to work in compartments that selectively attach different kinds of cellular population, for example by bioprinting different porosities/concentrations as demonstrated by Holmes et al. for bone reconstruction (Holmes et al., 2016). Given the promising results of GBM on stem cells, it is surprising that there is no clinical translation of research findings. *In vivo* tests of GBM regeneration of muscle are limited to the implantation of PCEG containing rGO that didn’t induce immune response (Du et al., 2018). However, this lack of immunogenicity cannot be generalized to all GBM materials: toxicity evaluation is made difficult by the infinite combinations of dose, shape, surface chemistry, exposure route and purity of graphenes used (Shareena et al., 2018). Future *in vivo* studies should foresee a strict application of guidelines to standardize the quality of toxicity evaluation (Reina et al., 2017). An ideal biomaterial, as well as matching native tissue compliance, should also degrade at a suitable rate to continue support during gradual reconstruction (4∼8 weeks after muscular trauma (Winkler et al., 2011)). Biocompatibility and biodegradation are certainly the Achille’s heel of GBM. GBM biodegradation is strictly dependent on the synthesis method, lateral size, and surface functionalization with polymers or proteins like albumin (Palmieri et al., 2019b; Syama and Mohanan, 2019). For this reason, it is difficult to generalize results on GBM degradability. However, it is ascertained that peroxidases or H_2_O_2_ alone can degrade graphene (Kotchey et al., 2011; Xing et al., 2014) and that the biodegradation of 3D scaffolds can be sped up by pretreatment with O_2_ plasma that increases hydrophilicity and number of defects (Loeblein et al., 2016). In conclusion, GBM promise to become the future exciting nanoplatforms for muscle engineering provided that nano-bio interactions and the toxic potential of GBM scaffold will be carefully evaluated.

## Author Contributions

All authors listed have made a substantial, direct and intellectual contribution to the work and approved it for publication.

## Conflict of Interest

The authors declare that the research was conducted in the absence of any commercial or financial relationships that could be construed as a potential conflict of interest.
